# Evaluation of changes in the epidemiology of leptospirosis in dogs after introduction of a quadrivalent antileptospiral vaccine in a highly endemic area

**DOI:** 10.1111/jvim.15947

**Published:** 2020-10-26

**Authors:** Thierry Francey, Ariane Schweighauser, Antonella Reber, Simone Schuller

**Affiliations:** ^1^ Division of Small Animal Internal Medicine, Department of Clinical Veterinary Medicine Vetsuisse Faculty University of Bern Bern Switzerland; ^2^ Washington State University Pullman Washington USA

**Keywords:** dog, immunization, infectious disease, zoonosis

## Abstract

**Background:**

Since 2003, a marked increase in leptospirosis serogroup Australis has been observed in dogs in Switzerland. In 2013, a new quadrivalent antileptospiral vaccine (L4) was introduced, adding serogroups Australis and Grippotyphosa to Canicola and Icterohaemorrhagiae of the previous bivalent vaccines (L2).

**Objective:**

To examine whether introduction of L4 was associated with decreased incidence of leptospirosis and decreased odds for dogs with acute kidney injury (AKI) to be diagnosed with leptospirosis.

**Animals:**

Four hundred and sixty‐nine dogs with AKI presented to a referral hospital, including 269 dogs with leptospirosis and 200 controls with other causes.

**Methods:**

Descriptive section: disease incidence was evaluated for 3 consecutive periods: before (PRE, 2011‐2012), transition (TRANS, 2013‐2014), and after introduction of L4 (POST, 2015‐2017). Analytical section: variables associated with a diagnosis of leptospirosis were investigated in a case‐control study using multivariable logistic regression, and focusing on vaccination.

**Results:**

The number of dogs diagnosed with leptospirosis (AKI‐L) decreased from 56.5 (PRE) to 15.7 (POST) cases/year while controls increased from 16.5 to 38.0 cases/year. Control dogs (AKI‐nL) showed a decrease in L2 vaccination (100% to 26%) and an increase in L4 vaccination (0% to 70%). The odds ratio for vaccinated dogs to be diagnosed with leptospirosis was 0.11 (95% confidence interval [CI], 0.06‐0.22; *P* < .001) for L4 and 2.08 (0.58‐7.42; *P* = .26) for L2.

**Conclusions and Clinical Importance:**

The introduction of L4 was associated with a marked decrease in dogs with leptospirosis and AKI in Switzerland. Use of the L4 vaccine was associated with significantly decreased odds of disease.

**Abbreviations**
AIC: Akaike Information Criterion
AKI: acute kidney injury
AKI‐L: acute kidney injury due to leptospirosis
AKI‐nL: acute kidney injury not due to leptospirosis
IQR: interquartile range
L: leptospirosis
L2: bivalent antileptospiral vaccine
L4: quadrivalent antileptospiral vaccine
MAT: microscopic agglutination test
OR: odds ratio
ROC‐AUC: area under the receiver operating characteristic curve
RT‐PCR: reverse transcriptase polymerase chain reaction

## 1 INTRODUCTION

Leptospirosis is a zoonotic disease with worldwide distribution affecting most mammalian species, including humans and dogs.^1^, ^2^ It is caused by infection with pathogenic *Leptospira* spp., which colonize the renal tubules of chronically infected hosts and are shed into the environment via urine. Maintenance hosts infected with host‐adapted *Leptospira* spp. typically are asymptomatic whereas incidental hosts can suffer a wide range of clinical manifestations including fever, kidney and liver injury, systemic and pulmonary hemorrhage, and reproductive failure.^3^, ^4^, ^5^, ^6^, ^7^ Dogs have been known to be susceptible to acute leptospiral infection for over 80 years,^8^ and serovars belonging to serogroups Canicola and Icterohaemorrhagiae have been thought until recently to be the main cause of clinical leptospirosis in dogs.^5^ Bivalent whole cell vaccines including serovars belonging to serogroups Canicola and Icterohaemorrhagiae have been available in Europe since the 1960s.^9^ These bivalent vaccines confer only partial or no immunity to heterologous serogroups such as Australis, Grippotyphosa and Pomona, which have been shown to cause acute disease in dogs.^10^, ^11^

After approximately 30 years of only occasional disease in Switzerland, the period between 2003 and 2012 was characterized by a 25‐fold increase in the number of cases of clinically manifested acute leptospirosis in dogs diagnosed at a veterinary teaching hospital, exceeding the incidence in other European countries.^4^ The main clinical manifestations of this re‐emerging epidemic of leptospirosis in dogs in Switzerland often were severe and included acute kidney injury (AKI, 99%), acute liver injury (26%), hemorrhagic tendencies (18%), and leptospiral pulmonary hemorrhage syndrome (77%). Approximately 70% of dogs in this cohort showed serologic evidence of infection with serovars Bratislava and Australis, both belonging to serogroup Australis.^4^

In 2013, a new quadrivalent antileptospiral vaccine (Nobivac Lepto 6, MSD Animal Health, also known as Nobivac L4 in other countries; L4) was introduced onto the Swiss market.^12^ This killed whole cell vaccine includes serovars of serogroups Australis and Grippotyphosa in addition to serogroups Canicola and Icterohaemorrhagiae present in the previously available bivalent vaccines (L2). The vaccine had been shown to provide excellent protection against experimental challenge of dogs with heterologous strains from the same serogroups.^12^ Because of effective marketing and continuous education efforts by specialists working in academia, the uptake of this vaccine in Switzerland appeared to be quick, providing the unique opportunity to study the change in epidemiology of leptospirosis in dogs associated with the change in prevention.

Therefore, the aims of our retrospective case‐control study were to describe the changes in numbers of cases of AKI in dogs with causal evidence of leptospirosis (AKI‐L) over a 7‐year period (2011‐2017) compared to a control group of similarly affected dogs with AKI not caused by leptospirosis (AKI‐nL), and to investigate vaccination‐related variables associated with a diagnosis of leptospirosis.

## 2 MATERIALS AND METHODS

### 2.1 Study design

The study was divided into 2 sections. The descriptive section was designed to report the incidences of AKI‐L and AKI‐nL in dogs presented to a veterinary teaching hospital. The incidences were evaluated in 3 consecutive time periods, spanning 2 years before introduction of the L4 vaccine (PRE, 2011‐2012), a transition period of 2 years during progressive uptake of the vaccine by practitioners (TRANS, 2013‐2014), and a 3‐year postintroduction period (POST, 2015‐2017). An analytical section was designed as a retrospective case‐control study to evaluate variables associated with a diagnosis of leptospirosis, focusing on antileptospiral vaccination status.^13^ For the case‐control study, a case was defined as a dog diagnosed with AKI caused by leptospirosis (AKI‐L). A control was defined as a dog diagnosed with AKI of other causes (AKI‐nL). This control population was chosen because it consists of dogs with similar clinical presentation and disease severity diagnosed during the same time.

### 2.2 Case definitions, diagnoses, inclusion, and exclusion criteria

All dogs diagnosed with AKI at the referral Small Animal Clinic of the Vetsuisse Faculty University of Bern between 2011 and 2017 were included in the study if they met the diagnostic criteria for either leptospirosis (AKI‐L) or control (AKI‐nL). Dogs presented for >1 episode of AKI only were included the first time. This total population of dogs diagnosed with AKI‐L and AKI‐nL formed the basis for the description of the disease and of the case numbers, independently of the availability of vaccination information. Only dogs with a complete vaccination history available from the medical records or retrievable retrospectively by contacting the owners or the referring veterinarians were included in the evaluation of a potential association between antileptospiral vaccination status and diagnosis of AKI‐L.

Acute kidney injury was defined by the combination of historical, clinical, laboratory, and imaging evidence, with at least 2 of the following criteria^14^: (a) presence of renal azotemia with a serum creatinine concentration ≥ 1.7 mg/dL persisting at least 24 hours after correction of prerenal factors; (b) increase in serum creatinine concentration ≥0.3 mg/dL during a 48‐hour  interval in the absence of prerenal factors; (c) persistent pathological oligo‐anuria (<1 mL/kg/h over 6 hours) after volume repletion; (d) and evidence of tubular injury with renal glucosuria or granular casts on urinalysis. Dogs with evidence of underlying chronic kidney disease were not excluded from the study, as long as they fulfilled the criteria for AKI.

### 2.3 Acute kidney injury due to leptospirosis (AKI‐L)

A suspicion of leptospirosis was based on compatible clinical findings indicative of an inflammatory syndrome (fever, muscle pain, reluctance to move), gastrointestinal disturbances (anorexia, abdominal pain, vomiting, diarrhea), hemostatic disturbances (petechiae, ecchymoses), renal involvement (oligo‐anuria, polyuria, fluid disturbances, hematuria, uremic odor, oral ulcerations), liver involvement (icterus), or pulmonary involvement (tachypnea, dyspnea, increased lung sounds including crackles). The suspicion was confirmed by the presence of at least 1 of the following, in order of preference: positive urine or tissue real‐time reverse transcriptase polymerase chain reaction (RT‐PCR) 1a); a microagglutination test (MAT) seroconversion with at least 4‐fold titer increase (1b); a single positive MAT titer ≥1:800 (1c); a single positive IgM lateral flow assay (1d); strong clinical evidence with at least 3 of the 4 classical organ manifestations of leptospirosis (renal, hepatic, pulmonary, hemorrhagic; 1e); or suggestive clinical evidence with 2 of the 4 organ manifestations (1f) in the absence of another cause. The last 2 criteria (1e and 1f) only were considered in cases in which leptospirosis could not be confirmed serologically because of early death.

### 2.4 Acute kidney injury not due to leptospirosis (AKI‐nL)

Control dogs were diagnosed and confirmed with AKI‐nL based on a known alternative diagnosis (2a) or a suspicion of a cause other than leptospirosis with negative paired MAT serology (2b). A tentative diagnosis of AKI‐nL was considered for dogs with a suspicion of AKI‐nL and 1 negative MAT serology (2c), and for dogs without evidence of leptospirosis in which no cause was positively identified (2d).

### 2.5 Confirmatory testing

The MAT testing was performed by the National Reference Laboratory for Leptospirosis, Institute of Veterinary Bacteriology, Vetsuisse Faculty University of Bern and was conducted using a panel of 12‐13 serovars, including *L. interrogans* serovars Australis, Autumnalis, Bataviae, Bratislava, Canicola, Copenhageni (added to the panel in 2015), Hardjo, Icterohaemorrhagiae, Pomona, Pyrogenes, Sejroe, and Tarassovi, and *L. kirschneri* serovar Grippotyphosa.

### 2.6 Vaccine use and vaccination status

Antileptospiral vaccination records were considered complete if they included the type of vaccine used (L2 or L4), as well as documentation of the initial vaccination consisting of 2 injections, of the individual boosters, and of the last vaccine. Lack of vaccination was differentiated from lack of recording by a thorough review of the dog's health maintenance booklet in which all vaccines are officially recorded. When a gap in health maintenance was observed, it was double‐checked by consulting the medical records of the referring veterinarian or by interviewing the dog's owners. Dogs with incomplete or doubtful records were excluded from the analyses.

Vaccine use was defined as the last vaccine type used in a defined dog before AKI diagnosis. In this respect, dogs were classified as either nonvaccinated (L0) if they had never received any antileptospiral vaccine, or vaccinated with either a bivalent vaccine (L2, serogroups Canicola and Icterohaemorrhagiae) or a quadrivalent vaccine (L4, serogroups Canicola, Icterohaemorrhagiae, Australis, and Grippotyphosa). Although dogs vaccinated with L4 technically also were immunized against the 2 serovars contained in L2, these dogs were considered L4 and not L2 vaccinated.

Based on their antileptospiral vaccination status, dogs were classified in 1 of 5 categories: nonvaccinated (L0), partially vaccinated with L2 (L2−), partially vaccinated with L4 (L4−), adequately vaccinated with L2 (L2+) or adequately vaccinated with L4 (L4+). The L2 and L4 vaccination status was considered adequate (L2+ or L4+) if the dogs had received the initial 2 injections at an interval of 20‐30 days, followed by yearly boosters (maximum interval of 18 months), and if the last injection was at most 365 days before the first clinical signs attributable to leptospirosis or AKI. Dogs were considered vaccinated with claim for protection starting 10 days after completion of the initial 2 injections. These criteria were based on pathophysiological justification of the expected immune response, clinical observations of humans and dogs with naturally occurring disease, and unpublished observations from the vaccine manufacturer.^2^, ^12^ When ≥1 of these criteria were not fulfilled in vaccinated dogs, vaccination status was considered partial or inadequate (L2− or L4−). Dogs vaccinated correctly or partially with L4 were considered L4+ or L4−, respectively, even if they previously had been vaccinated correctly with L2. Dogs never vaccinated with any antileptospiral vaccine (L2 or L4) were classified as L0.

For the analysis related to the L4 vaccination status, all dogs were classified as L0, L4−, or L4+. Similarly, for the analyses related specifically to the L2 vaccination status, all dogs never vaccinated with L4 were classified as L0, L2−, or L2+.

Dogs correctly vaccinated with either L2 or L4 and diagnosed with acute leptospirosis were classified as cases of leptospirosis in L2 or L4 vaccinated dogs, respectively. Age, diagnostic criteria, seropositivity rates, and time from last vaccination were reviewed separately for these cases of particular interest in the context of vaccine protection.

### 2.7 Statistical analysis

General: Sample size calculations and data analyses were performed using commercial software programs (PASS 13 Power Analysis and Sample Size Software, 2014 and NCSS 9 Statistical Software, 2013; NCSS, LLC. Kaysville, Utah, respectively).

The continuous variables age and time from vaccination were tested for normality by visual inspection of histograms and a Shapiro Wilk test, both for all dogs with AKI and for the subgroups, when applicable. Because they were not normally distributed, their descriptive statistics were reported as median (interquartile range, IQR) and they were compared among groups using a Kruskal‐Wallis one‐way analysis of variance (ANOVA). Categorical variables were reported as absolute numbers and proportions, and were compared among groups using a chi‐square test or a Fisher's exact test when the expected counts were <5 in a cell from the contingency tables. They included variables compared among time periods (PRE, TRANS, POST): diagnosis, availability of complete vaccination information, presence of adequate vaccination status (L2, L4, or any L‐vaccine), and MAT seropositivity for 13 individual serovars. Variables compared among the categories of vaccination status (L0, L4−, L4+) were: sex, neuter status, and breed groups, and variables compared between the 2 disease groups (AKI‐L, AKI‐nL) were: sex (male, female), neuter status (intact, neutered), breed group (herding, hound, nonsporting, sporting, terrier, toy, working, and mixed breed), organ manifestations (hepatic, hemostatic, pulmonary), vaccine use (L0, L2, L4), and vaccination status (L0, L2−, L2+, L4−, L4+; adequate status for any L‐vaccine; adequate protection for 2 serogroups; adequate protection for 4 serogroups). Unless mentioned otherwise, a *P*‐value of .05 was used as cut‐off for statistical significance.

Descriptive section: Average annual case numbers, vaccine use, and vaccination status were calculated for both disease groups for the 3 study periods: PRE, 2011‐2012; TRANS, 2013‐2014; POST, 2015‐2017. Yearly case numbers were calculated based on the total population of dogs diagnosed with AKI‐L and AKI‐nL, independently of whether vaccination information was available or not. Vaccine use, vaccination status, and associations between vaccination and disease were assessed in the subpopulation of dogs with known vaccine information.

Analytical section: The association between vaccination status and diagnosis of leptospirosis was evaluated using multivariable logistic regression analysis as the odds ratio (OR) for a dog with AKI to be diagnosed with AKI‐L vs AKI‐nL, if correctly vaccinated (L2+, L4+) or partially vaccinated (L2−, L4−) vs not vaccinated (L0 as reference).^15^ Separate analyses were performed for L2 and L4 vaccination status, because too many combinations and therefore subgroups would have been necessary to cover all possible status variations with a single model. Because of common changes among different brands of L2 used within individual dogs, the effect of L2 was not evaluated at the brand level but as a group effect. Assuming that antileptospiral vaccination neither caused nor protected from AKI‐nL, this OR can be viewed as an estimation of the association of vaccination with disease.^16^

An estimation of the sample size necessary for the planned analyses was performed with the following variables and assumptions, based on a preliminary evaluation at our institution: L4 vaccination status (L4+, L4−, L0 [reference]) as independent variable; diagnosis (AKI‐L, AKI‐nL [reference]) as binary dependent variable; assumption of a change in the probability for a dog with AKI to be diagnosed with AKI‐L from 75% when not vaccinated (estimated from the hospital population, 2011‐2012) to <33% when vaccinated with L4 (estimated from the hospital population, 2015‐2017); proportion of dogs current on their antileptospiral vaccination 50%; power 0.9 and alpha 0.05. This analysis yielded a minimal number of 54 dogs with AKI necessary for univariable logistic regression analysis. The addition of confounders with low correlation to the independent variable in a multivariable logistic regression model would require 109 dogs with AKI.

A similar estimation was performed for L2 vaccination status, using the assumption that a change in the proportion of AKI diagnosis from 75% when not vaccinated (estimated from the hospital population, 2011‐2012) to 50% for AKI‐L (a decrease of at least 33% was considered arbitrarily to be necessary to be considered clinically relevant), when correctly vaccinated with L2, should be recognized. It yielded a minimum number of 153 dogs with AKI for univariable analysis and 306 dogs with AKI when confounders were included in a multivariable logistic regression model.

Sex, neuter status and breed group have been reported to be associated with both the independent and the dependent variables.^4^, ^17^, ^18^, ^19^ They therefore were assessed as potential confounders by evaluating their statistical associations with both the independent and dependent variables: sex (female vs male), neuter status (neutered vs intact), breed group (herding, hound, nonsporting, sporting, terrier, toy, working, and mixed breed), and age (as a continuous variable). Variables with *P* values <.10 (Kruskal‐Wallis or chi‐square test) were considered potentially associated with the independent, dependent or both variables and were included as potential confounders in the multivariable analysis. These variables were added 1‐by‐1 in the multivariable logistic regression model (log[OR] = *β*_1_ + *β*_2_ * [vaccination status] + *β*_i_ * [confounder_i_], including interactions), based on their log‐likelihood, using a forward variable selection approach.^15^ Variables were included until the addition of further variables did not result in a better‐fit model, indicated by a lack of a further decrease of the Akaike Information Criterion (AIC) value. The following diagnostics were conducted on the obtained logistic regression models: continuous variables of the models were evaluated for linearity by visual evaluation of the plot of their relationship with the logit of outcome; the included variables and their interactions were evaluated by introducing them in the models and checking their effect on the regression coefficient *β* of the remaining variables (a change >20% was considered significant to keep them in the model); the model fit was assessed using the Pearson chi‐square goodness‐of‐fit statistic and outliers were identified by analyzing the Pearson residuals (<−2 or >2). The percentage of correct predictions and the area under the receiver operating characteristic curve (ROC‐AUC) were calculated as additional indicators of model performance.

To minimize potential misclassification bias, both logistic regression analyses were repeated using a restricted case definition after excluding cases and controls diagnosed based on clinical evidence only (1e, 1f, 2d).

## 3 RESULTS

### 3.1 Dogs and diseases

**FIGURE 1:**
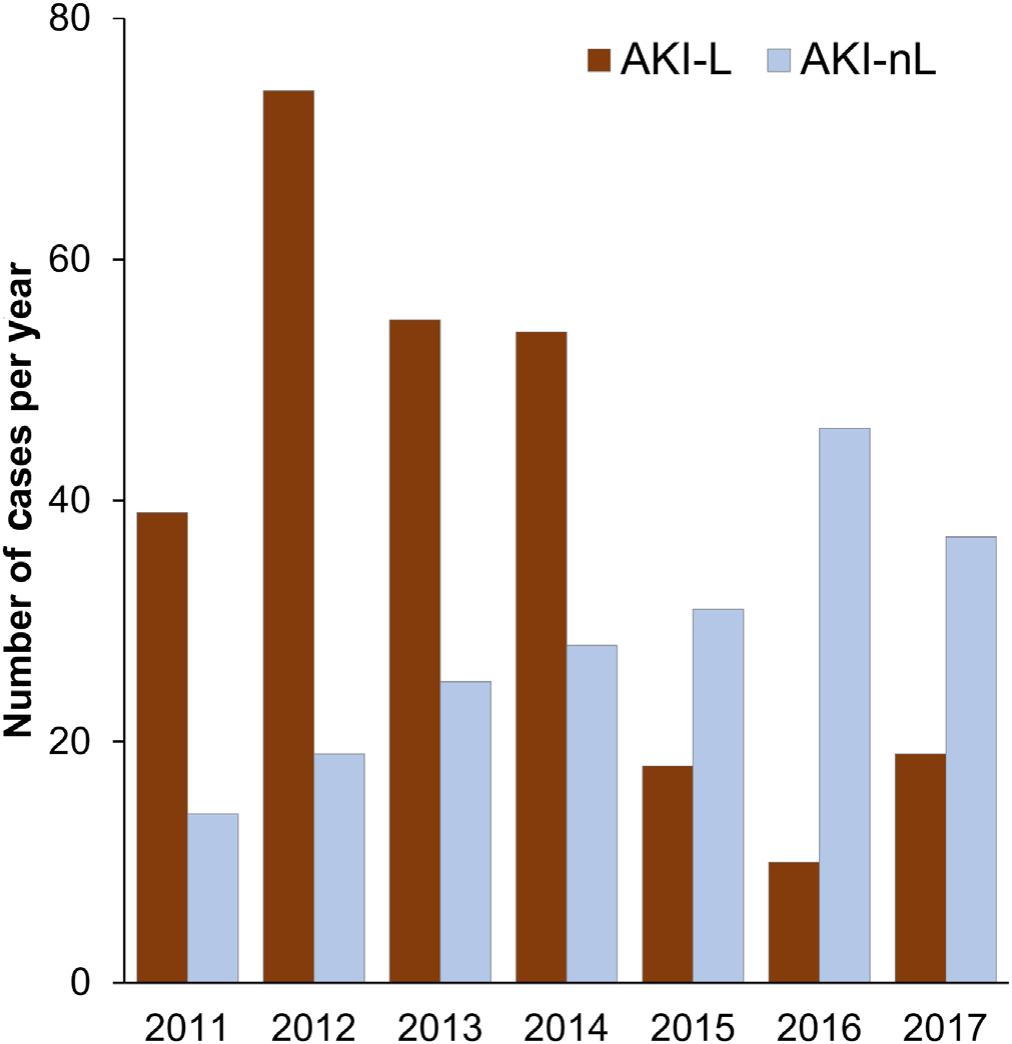
Yearly incidence of diagnosis of leptospirosis (AKI‐L) and of AKI not caused by leptospirosis (AKI‐nL) during the 7 years of the study spanning before and after the introduction of the new L4 vaccine in 2013

During the study period from 2011 to 2017, 469 dogs were diagnosed with AKI‐L (n = 269) or AKI‐nL (n = 200). Complete vaccination information was available for 368 dogs (78%): 223 dogs with AKI‐L (83%) and 145 dogs with AKI‐nL (73%). The proportion of dogs with complete vaccination information was higher in dogs with AKI‐L than in those with AKI‐nL (*P* = .007), but unchanged among the 3 time periods (PRE, 79%; TRANS, 77%; POST, 80%; *P* = .92). The numbers of dogs with a diagnosis of AKI‐L and AKI‐nL included for each year of the study are shown in Figure 1. Of these, 115 dogs were available with complete vaccination information in time period PRE (97, AKI‐L; 18, AKI‐nL), 125 in time period TRANS (87, AKI‐L; 38, AKI‐nL), and 128 in time period POST (39, AKI‐L; 89, AKI‐nL). The demographics and main disease characteristics of both study groups are summarized in Table 1. The average number of dogs diagnosed with AKI‐L was significantly lower in the POST period than in earlier time periods (*P* < .0001) and the number of dogs with AKI‐nL was significantly higher in the POST period than in earlier time periods (*P* < .0001).

**TABLE 1:** Number of cases, demographics, main disease characteristics, and basis for diagnosis of 469 dogs with AKI due to leptospirosis (AKI‐L) or other causes (AKI‐nL)

		AKI‐L	AKI‐nL
All dogs with diagnosis		269	200
Dogs with complete vaccination history (% of all dogs)		223 (83%)	145 (73%)
Number of cases (average per year)			
PRE (2011‐2012)		56.5	16.5
TRANS (2013‐2014)		54.5	26.5
POST (2015‐2017)		15.7	38.0
Sex			
Female [intact/spayed]		92 (34%) [32/60]	79 (40%) [22/57]
Male [intact/castrated]		177 (66%) [114/63]	121 (61%) [65/56]
Age (y) (median, IQR)		5.9 (1.8‐8.7)	7.3 (4.1‐10.6)
Main organ manifestations			
Renal		269 (100%)	200 (100%)
Hepatic		102 (38%)	37 (19%)
Pulmonary		165 (61%)	16 (8%)
Hemorrhagic		106 (39%)	56 (28%)
Basis for diagnosis			
PCR	(1a)	6 (2%)	
MAT seroconversion	(1b)	123 (46%)	
Single MAT	(1c)	81 (31%)	
Rapid test	(1d)	25 (9%)	
Strong clinical evidence	(1e)	26 (10%)	
Suggestive clinical evidence	(1f)	8 (3%)	
Established diagnosis	(2a)		156 (78%)
Suspected diagnosis + MAT 2× neg	(2b)		13 (7%)
Suspected diagnosis + MAT 1× neg	(2c)		81 (31%)
No evidence of leptospirosis	(2d)		14 (7%)

Abbreviations: AKI, acute kidney injury; IQR, interquartile range; L, leptospirosis; MAT, microscopic agglutination test; MAT 2x neg, negative paired MAT serology; MAT 1× neg, 1 negative MAT serology; nL, nonleptospirosis; PRE, period before the introduction of L4; POST, period after the introduction of L4; PCR, polymerase chain reaction; TRANS, transition period.

The basis for the diagnosis of dogs as AKI‐L and AKI‐nL is described in Table 1. The repartition of the categories was almost identical in the subgroup of 223 dogs with available vaccine information as for the whole group. Seropositivity with a MAT titer ≥1:800 was detected for the following serovars: Australis (73%), Bratislava (64%), Copenhageni (29%), Pomona (28%), Autumnalis (26%), Grippotyphosa (15%), Canicola (9%), Icterohaemorrhagiae (5%), Pyrogenes (1%), and Bataviae (1%). The prevalence of seropositivity increased significantly over time for serovar Bratislava (54% PRE to 77% POST, *P* = .01), with no change for the other serovars.

Diagnoses identified in the group AKI‐nL included toxicoses (n = 49, 25%), infections (n = 27, 14%), inflammatory diseases (n = 11, 6%), hemodynamic disturbances (n = 14, 7%), multisystemic diseases (n = 70, 35%), acute‐on‐chronic kidney decompensation (n = 11, 6%), upper urinary tract obstruction (n = 2, 1%), and unclear etiology (n = 16, 8%). *Leptospira* seropositivity with a MAT titer ≥1:800 was not observed in dogs with AKI‐nL. Using a lower cutoff of 1:100, seropositivity in these dogs was detected for the following serovars: Canicola (12%), Copenhageni (8%), Australis (6%), Grippotyphosa (4%), Bratislava (3%), Pomona (3%), and Icterohaemorrhagiae (3%). No obvious difference was noted between vaccinated and nonvaccinated dogs for the corresponding serovars, but no statistical comparison was performed because of the low number of dogs in some groups. Dogs with AKI‐nL were significantly less likely to have hepatic (*P* < .0001), hemorrhagic (*P* = .01) or pulmonary (*P* < .0001) involvement than were dogs with AKI‐L.

### 3.2 Vaccine use and vaccination status

**FIGURE 2:**
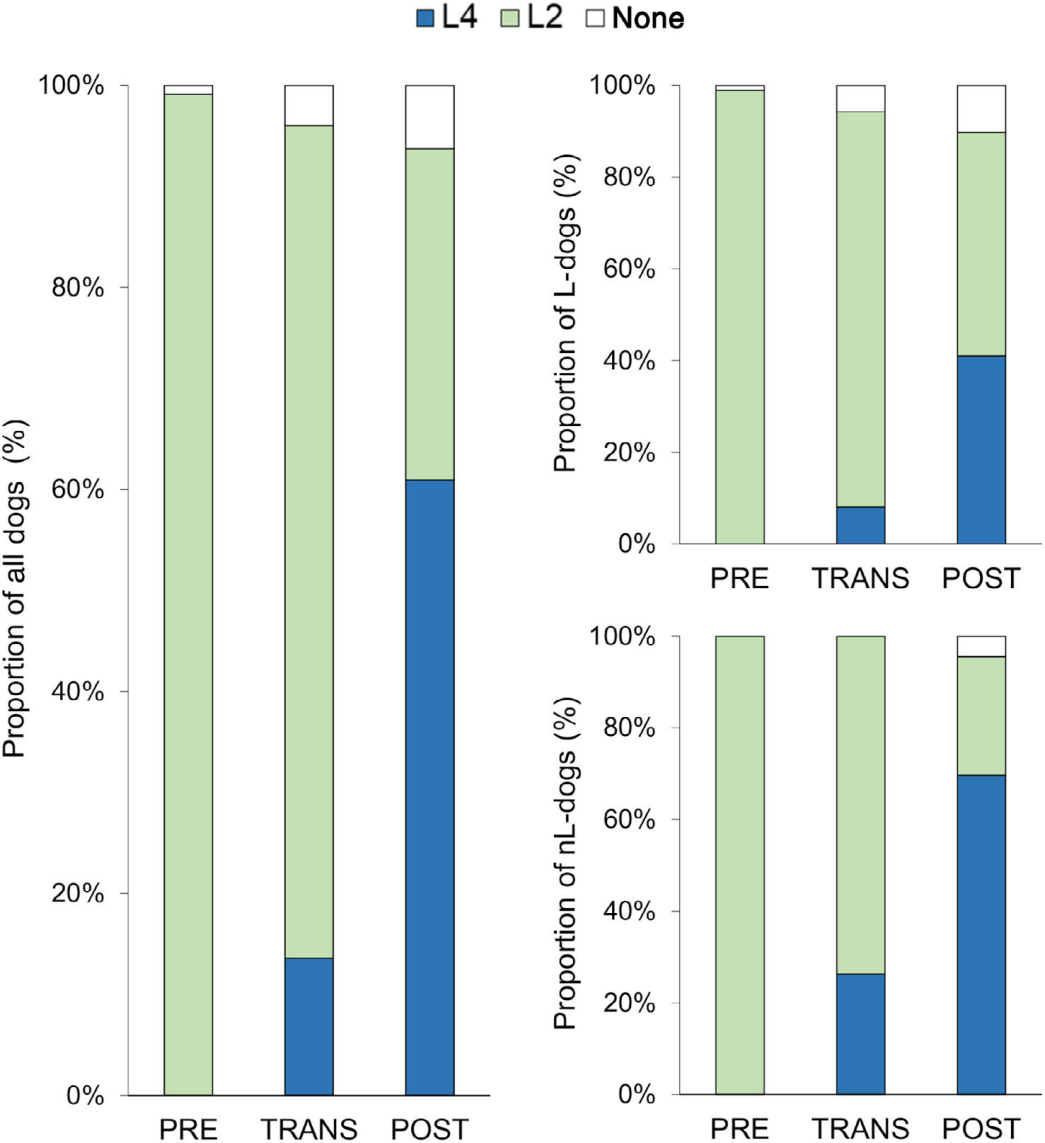
Change in vaccine use in 368 dogs with AKI (left panel), including 223 dogs with AKI‐L (top right panel) and 145 dogs with AKI‐nL (low right panel). Dogs were classified as nonvaccinated (L0), vaccinated with L2, or vaccinated with L4, and grouped according to the 3 time periods of the study

**FIGURE 3:**
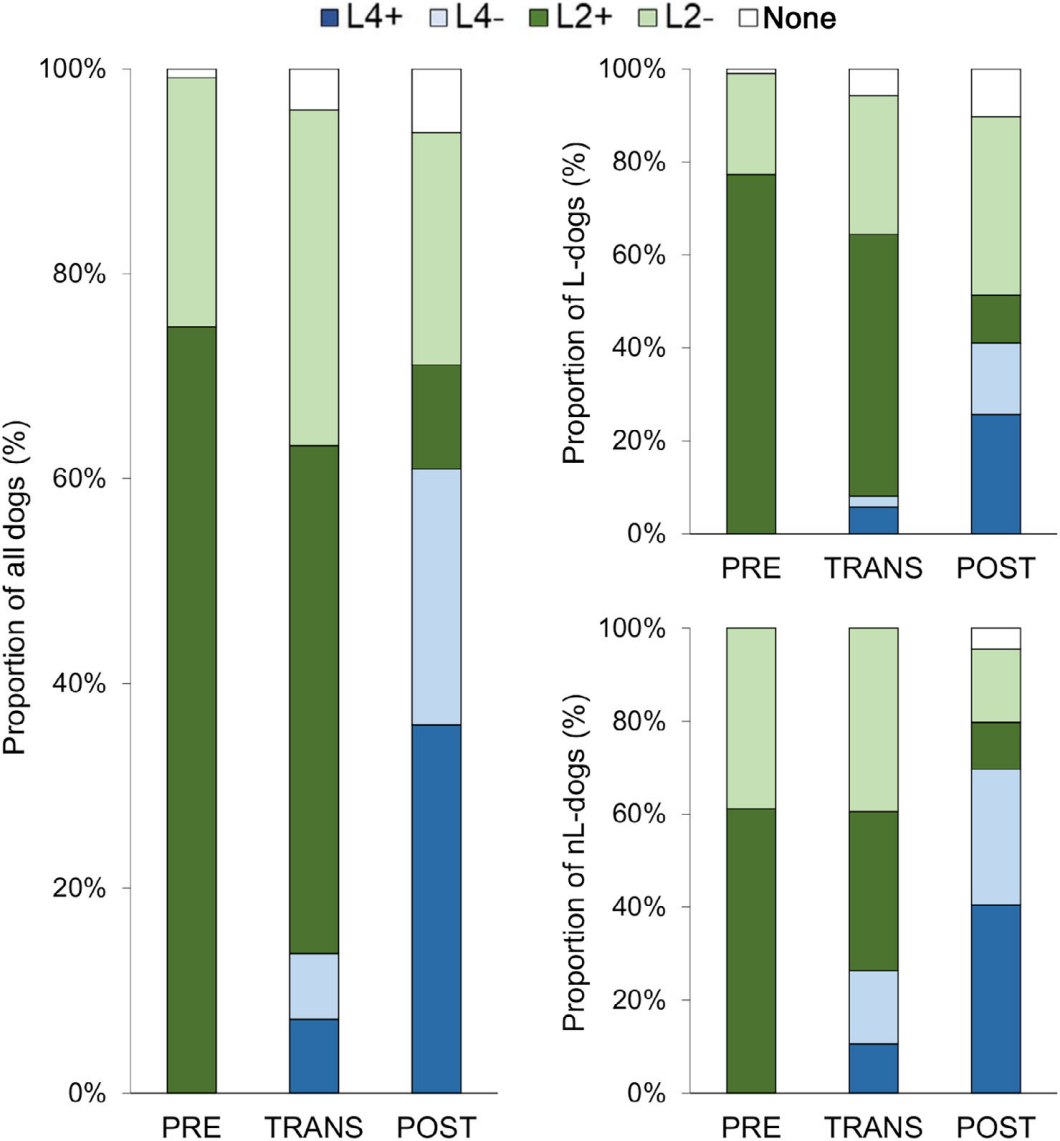
Vaccination status of 368 dogs with AKI (left panel), including 223 dogs with AKI‐L (top right panel) and 145 dogs with AKI‐nL (low right panel). Dogs were classified as nonvaccinated (L0), correctly vaccinated with L2 (L2+), inadequately vaccinated with L2 (L2−), correctly vaccinated with L4 (L4+), or inadequately vaccinated with L4 (L4−), and they were grouped according to the 3 time periods of the study

Data on vaccine use and vaccination status are summarized in Table 2, and changes over time are represented in Figures 2 and 3. The proportion of the control AKI‐nL dogs never vaccinated remained low throughout the study (0/18 PRE, 0/38 TRANS, and 4/89 POST). The partial immunization status for L4 observed in 32 dogs was because of incorrect primary vaccination (n = 25), incorrect yearly booster schedule (n = 7), or a last vaccine administration >365 d before presentation for AKI (n = 14); >1 condition was not fulfilled in 10 dogs.

**TABLE 2:** Vaccine use and vaccination status in dogs with AKI due to leptospirosis (AKI‐L) or other causes (AKI‐nL)

	AKI‐L (n = 223)	AKI‐nL (n = 145)	*P* (L vs nL)
Vaccine use at the time of disease			
L0	10 (4%)	4 (3%)	.4
L2	190 (85%)	69 (48%)	<.001
L4	23 (10%)	72 (50%)	<.001
Vaccination status			
L0	10 (4%)	4 (3%)	.4
L2−	62 (28%)	36 (25%)	.53
L2+	128 (57%)	33 (23%)	<.001
L4−	8 (4%)^a^	32 (22%)^b^	<.001
L4+	15 (7%)	40 (28%)	<.001
Current protection for 2 SG at the time of diagnosis^c^	147 (66%) PRE 75/97 (77%) TRANS 56/87 (64%) POST 16/39 (41%)	87 (60%) PRE 11/18 (61%) TRANS 21/38 (55%) POST 55/89 (62%)	.2
Current protection for 4 SG at the time of diagnosis^d^	15 (7%) PRE 0/97 (0%) TRANS 5/87 (6%) POST 10/39 (26%)	40 (28%) PRE 0/18 (0%) TRANS 4/38 (11%) POST 36/89 (40%)	< .001
Partial protection for 2 SG at the time of diagnosis^c^	66 (30%) PRE 21/97 (22%) TRANS 26/87 (30%) POST 19/39 (49%)	54 (37%) PRE 7/18 (39%) TRANS 17/38 (45%) POST 30/89 (34%)	= .13
Partial protection for 4 SG at the time of diagnosis^d^	8 (4%) PRE 0/97 (0%) TRANS 2/87 (2%) POST 6/39 (15%)	32 (22%) PRE 0/18 (0%) TRANS 6/38 (16%) POST 26/89 (29%)	< .001

*Note: P* refers to the statistical significance of the comparisons of proportions between the groups L and nL, using a chi‐square test. The comparisons in the lower part of the table refer only to the proportions calculated for the whole study and not for the individual time periods.

Abbreviations: AKI, acute kidney injury; L, leptospirosis; nL, nonleptospirosis; L0, never vaccinated with an antileptospiral vaccine; L2, bivalent vaccine; L4, quadrivalent vaccine; L2−, vaccinated with L2 but not current on L2; L2+ current on L2; L4−, vaccinated with L4 but not current on L4; L4+ current on L4; PRE, period before the introduction of L4; POST, period after the introduction of L4; SG, serogroup.

^a^ 4 of these 8 AKI‐L dogs (L4−) had ongoing appropriate protection for the 2 serogroups Canicola and Icterohaemorrhagiae, as the L2 vaccine was just replaced with L4 without new primo‐vaccination.

^b^ 14 of these 32 AKI‐nL dogs (L4−) had ongoing appropriate protection for the 2 serogroups Canicola and Icterohaemorrhagiae, as the L2 vaccine was just replaced with L4 without new primo‐vaccination.

^c^ Serogroups Canicola and Icterohaemorrhagiae.

^d^ Serogroups Canicola, Icterohaemorrhagiae, Australis, and Grippotyphosa.

### 3.3 Logistic regression analysis

Based on sample size analysis, the study population of 368 dogs with complete vaccination information was considered sufficient to address the study aims, including the evaluation of confounder variables. The following variables were evaluated as potential confounders for the logistic regression analysis: sex, neuter status, breed group, and age (Tables 3 and 4). Sex and breed group were eliminated because they were not associated with both the independent and dependent variables. Neuter status was associated with the dependent variable and age with both the independent and dependent variables. They both were included in the multivariable models.

**TABLE 3:** Descriptive statistics of the variables evaluated as potential confounders for the logistic regression analysis evaluating the association of L4 vaccination status and a diagnosis of leptospirosis

	AKI‐L (N = 223)	AKI‐nL (N = 145)	*P*
Age (years) [median, IQR]	5.7 (1.6‐8.7)	7.5 (4.1‐10.8)	<.001
Sex			
Female	75 (34%)	54 (37%)	.48
Male	148 (66%)	91 (63%)	
Neuter status			
Intact	123 (55%)	64 (44%)	.04
Neutered	100 (45%)	81 (56%)	
Breed group			.6
Herding	30 (13%)	25 (17%)	
Hound	11 (5%)	5 (3%)	
Nonsporting	13 (6%)	11 (8%)	
Sporting	68 (30%)	34 (23%)	
Terrier	21 (9%)	9 (6%)	
Toy	21 (9%)	15 (10%)	
Working	28 (13%)	20 (14%)	
Mixed breed	31 (14%)	26 (18%)	

*Note*: The 368 dogs diagnosed with AKI for which complete vaccination information was available were stratified based on their diagnosis. *P* refers to the statistical significance of a Kruskal‐Wallis one‐way ANOVA (continuous variable: age) or of the chi‐square test (categorical variables: sex, neuter status, breed group).

Abbreviations: AKI‐L, acute kidney injury due to leptospirosis; AKI‐nL, acute kidney injury from other causes than leptospirosis; IQR, interquartile range.

**TABLE 4:** Descriptive statistics of the variables evaluated as potential confounders for the logistic regression analysis evaluating the association of the L4 vaccination status with a diagnosis of leptospirosis

	L0 (*N* = 55)	L4− (*N* = 40)	L4+ (*N* = 273)	*P*
Age (years) [median, IQR]	6.4 (2.5‐9.1)	7.7 (3.5‐11.3)	5.9 (1.6‐8.9)	.09
Sex				
Female	94 (34%)	17 (43%)	18 (33%)	.56
Male	179 (66%)	23 (58%)	37 (67%)	
Neuter status				
Intact	139 (51%)	20 (50%)	28 (51%)	.99
Neutered	134 (49%)	20 (50%)	27 (49%)	
Breed group				.95
Herding	40 (15%)	8 (20%)	7 (13%)	
Hound	11 (4%)	1 (3%)	4 (7%)	
Nonsporting	18 (7%)	3 (8%)	3 (5%)	
Sporting	77 (28%)	9 (23%)	16 (29%)	
Terrier	23 (8%)	4 (10%)	3 (5%)	
Toy	29 (11%)	4 (10%)	3 (5%)	
Working	34 (12%)	4 (10%)	10 (18%)	
Mixed breed	41 (15%)	7 (18%)	9 (16%)	

*Note*: The 368 dogs diagnosed with AKI for which complete vaccination information was available were stratified based on their L4‐vaccination status. *P* refers to the statistical significance of the Kruskal‐Wallis one‐way ANOVA (continuous variable: age) or of the chi‐square test (categorical variables: sex, neuter status, breed group).

Abbreviations: L0, not vaccinated with L4; L4−, partially vaccinated with L4; L4+, correctly vaccinated with L4; IQR, interquartile range.

In the L4 multivariable analysis, vaccination status and age were strongly associated with diagnosis (Table 5). Vaccinated dogs and older dogs were at lower odds to be diagnosed with leptospirosis than were nonvaccinated or younger dogs, respectively. The OR for a dog presented with AKI to be diagnosed with AKI‐L vs AKI‐nL was 0.112 (95% CI, 0.056‐0.222; *P* < .001) when correctly vaccinated with L4 (L4+) and 0.090 (0.039‐0.211; *P* < .001) when partially vaccinated with L4 (L4−). In the L2 multivariable analysis, only age and neuter status were significantly associated with the diagnosis but not L2 vaccination status (Table 5). The OR for a dog presented with AKI to be diagnosed with AKI‐L vs AKI‐nL was 2.078 (0.582‐7.419; *P* = .26) when correctly vaccinated with L2 (L2+) and 1.037 (0.286‐3.757; *P* = .96) when partially vaccinated with L2 (L2−). Diagnostics conducted on both models are reported in Table 5.

**TABLE 5:** Results of the multivariable logistic regression analyses for associations with a diagnosis of AKI‐L vs AKI‐nL as dependent variable

Variable (reference)	*β*	SE	OR	95% CI of OR	Wald‐*P*
*L4‐vaccination model*					
Intercept	1.70	0.28	5.493	3.175‐9.506	<.001
L4‐vaccination status (L0)					
L4+	−2.19	0.35	0.112	0.056‐0.222	<.001
L4−	−2.40	0.43	0.090	0.039‐0.211	<.001
Age	−0.13	0.03	0.877	0.824‐0.934	<.001
Neuter status (intact)					
Neutered	−0.48	0.25	0.619	0.381‐1.005	.05
73.4% dogs classified correctly; ROC‐AUC, 0.774 Pearson chi‐square 69.1; 12 outliers on Pearson residual analysis					

Variable (reference)	*β*	SE	OR	95% CI of OR	Wald‐*P*
*L2‐vaccination model*					
Intercept	0.72	0.64	2.046	0.588‐7.120	.26
L2‐vaccination status (L0)					
L2+	0.73	0.65	2.078	0.582‐7.419	.26
L2−	0.04	0.66	1.037	0.286‐3.757	.96
Age	−0.10	0.04	0.905	0.839‐0.976	.009
Neuter status (intact)					
Neutered	−0.85	0.29	0.426	0.239‐0.759	.004
61.2% dogs correctly classified; ROC‐AUC, 0.690					
Pearson chi‐square 119.3; 1 outlier on Pearson residual analysis					

*Note*: These analyses include all 368 dogs with complete vaccination information for the L4 model and 273 dogs for the L2 model. Additional diagnostics conducted on both models include the linearity check for the continuous variable age. The variable checks confirmed the inclusion of the model variables except for the L2‐vaccination status in the L2‐model; none of the variable interactions was retained.

Abbreviations: *β*, regression coefficient; OR, odds ratio; 95% CI, 95% confidence interval; ROC‐AUC, area under the curve of the receiver operating characteristic curve.

The analyses based on the more restrictive case definition included 333 dogs for L4 (198 AKI‐L; 135 AKI‐nL) and 244 dogs for L2 (179 AKI‐L; 65 AKI‐nL). They gave very similar results as the models including all dogs with vaccination information (Table S1, Supporting Information). The L4 model indicated ORs of 0.081 (0.038‐0.174; *P* < .001) for L4+ and 0.091 (0.038‐0.214; *P* < .001) for L4−. The L2 model indicated ORs of 2.215 (0.610‐8.046; *P* = .23) for L2+ and 1.044 (0.285‐3.832; *P* = .95) for L2−. Both models included age and neuter status as significant covariates.

### 3.4 Leptospirosis in vaccinated dogs

**FIGURE 4:**
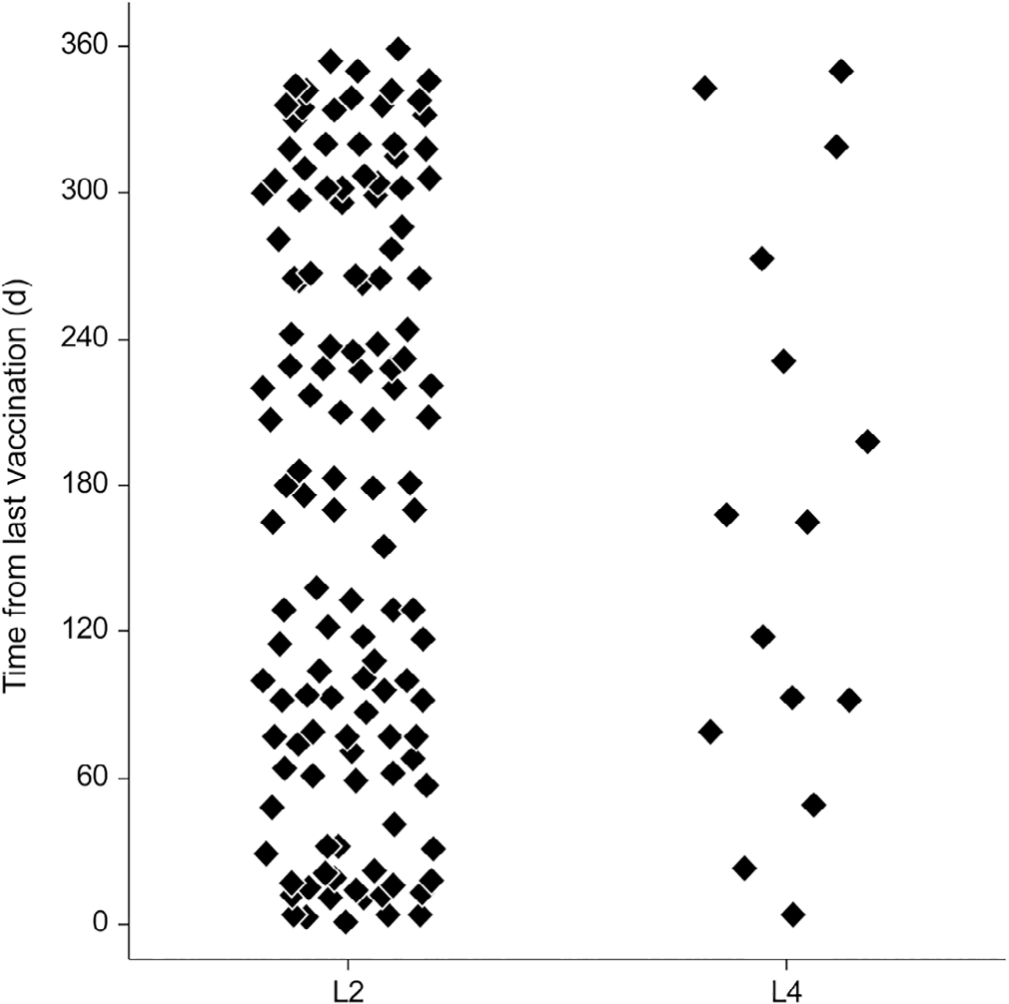
Time between the last antileptospiral vaccine administration and the first signs of leptospirosis in dogs correctly vaccinated with L2 (n = 128 dogs) or L4 (n = 15 dogs)

During the 7 years of the study (PRE, TRANS, POST), 128 cases of leptospirosis in L2‐vaccinated dogs were observed. Median age of the affected dogs was 5.8 years (IQR, 1.2‐8.8; range, 0.2‐15.3). All dogs had received a correct vaccination schedule. Median time from the last vaccination to the first signs of disease was 181 days (IQR, 76‐296; Figure 4), with no obvious clustering of cases in any of the postvaccination time periods. Seropositivity rates (MAT titer ≥1:800) were similar in correctly and incorrectly L2‐vaccinated dogs with AKI‐L: Australis (75%/73%), Bratislava (62%/71%), Copenhageni (22%/32%), Pomona (27% / 31%), Autumnalis (25%/29%), Grippotyphosa (19%/13%), Canicola (12%/7%), Icterohaemorrhagiae (5%/7%), Pyrogenes (1%/2%), and Bataviae (1%/1%).

During the last 5 years of the study (TRANS, POST), 15 cases of leptospirosis in L4‐vaccinated dogs were observed. Median age of the affected dogs was 1.1 years (IQR, 0.7‐2.8; range, 0.4‐13.6). All dogs had received a correct vaccination schedule. Thirteen dogs were affected within 1 year of the end of the initial primary vaccination and 2 dogs had received 1 booster vaccine afterward. Time from last vaccination to first clinical signs was a median of 165 days (IQR, 86‐252; Figure 4), with no obvious clustering of cases in any of the time periods. The diagnosis of leptospirosis was confirmed based on MAT seroconversion in 7 dogs (1b), a single positive MAT titer in 3 dogs (1c), a positive IgM lateral flow assay in 1 dog (1d), and strongly suggestive clinical signs with 3 of the 4 classical manifestations of leptospirosis in 4 dogs (1e). The prevalence of seropositivity for the 13 dogs with available serology was similar to other dogs with AKI‐L: Australis (77%), Bratislava (69%), Grippotyphosa and Autumnalis (23%), Pomona (15%), Copenhageni (25%), and Bataviae and Hardjo (8%). These dogs were similarly affected clinically as were other dogs with AKI‐L, including renal (n = 15, 100%), hepatic (n = 4, 27%), hemorrhagic (n = 5, 33%) and pulmonary (n = 9, 60%) manifestations.

During the last 5 years of the study (TRANS, POST), another 8 dogs not properly vaccinated with L4 were diagnosed with leptospirosis. The initial vaccination had not been performed correctly in 5 dogs (including 2 dogs missing the second injection because of AKI‐L and 3 dogs receiving only 1 injection), yearly boosters had been missed in 2 dogs, and the last vaccine administration was >365 days before presentation in 1 dog.

## 4 DISCUSSION

Between 2003 and 2012, Switzerland has experienced a steep increase in the incidence of acute leptospirosis in dogs, reaching epidemic proportions with a peak of 28.1 confirmed cases of severe disease per 100 000 dogs/year in the most affected area.^4^ During that time, infections in dogs were most commonly associated with serogroup Australis, suggesting that the then available bivalent antileptospiral vaccine did not confer sufficient protection against infection with this serogroup. In 2013, a new quadrivalent antileptospiral vaccine, including serogroups Australis and Grippotyphosa in addition to serogroups Canicola and Icterohaemorrhagiae was introduced to the Swiss market.^12^ The rapid increase in use of the L4 vaccine observed in our control group likely mirrors the uptake of the vaccine in the general dog population in Switzerland. This probably reflects the expectation of well‐informed owners and of veterinarians confronted with the clinical challenges of severe leptospirosis, hoping that the new quadrivalent vaccine would fill the protection gap left by the former bivalent vaccines.^4^ Interestingly, despite motivation to change vaccine type in 70% of the control dogs, only 40% of the controls were correctly vaccinated with L4 in the POST period, emphasizing continued need for veterinarian and owner education.

The number of cases of leptospirosis diagnosed at the Vetsuisse Faculty University of Bern decreased rapidly and sustainably from 2015 onwards whereas the number of dogs with AKI‐nL increased throughout the study period. This development shows that the decrease in leptospirosis cases was not because of a decreased nephrology caseload, but because of a selective decrease in cases of leptospirosis, indicating either a true decrease in the number of cases or a decrease in disease severity, no longer requiring specialized hospital care. At the same time, not only vaccine use but also actual vaccination status of the dogs included in the study changed from a bivalent to a predominantly quadrivalent vaccination, whereas the proportion of unvaccinated or incompletely vaccinated dogs remained stable over time. Because previously observed fluctuations in the number of cases of leptospirosis^4^ never reached this magnitude and were never sustained over 1 year, it is possible that the observed decrease in cases of leptospirosis in dogs is linked to the introduction of L4, although changes in the biology of the environment, in the disease pressure, or in the risk of pathogen exposure cannot be ruled out.

The timely association between introduction of the new vaccine and the marked decrease in disease incidence is particularly striking, considering that only 40% of the dogs were correctly immunized. Whether this outcome can be explained by apparent protection despite incomplete L4 vaccination, as suggested by these results, should be verified because of the very small size of some of the subgroups, especially the L4− dogs diagnosed with AKI‐L.

Lack of a mandatory national disease registry limits ability to evaluate major epidemiological changes for diseases such as leptospirosis and broad‐scale interventions such as vaccinations. Previous studies have reported the change in disease incidence associated with vaccination for various diseases in dogs, including rabies and leptospirosis.^20^, ^21^ However, ours is to the best of our knowledge among the few reports also documenting the detailed vaccination status of the affected and control dogs, and testing its association with the diagnosis. To do so, we chose a retrospective case‐control study design, based on a referral population from a single hospital, using dogs with AKI not caused by leptospirosis as controls. This very specific reference population only partially represents the general dog population in terms of vaccine use and vaccination status, and it is likely over‐represented by dogs from very motivated owners, ready to be referred and treated at a specialized institution. However, it is probably the most adequate control group for dogs with leptospirosis in terms of referral bias. Because >99% of dogs with leptospirosis in Switzerland are presented with AKI,^4^ most dogs ultimately diagnosed with leptospirosis were referred for evaluation and treatment of this condition. The differentiation between AKI‐L and AKI‐nL was in most cases established during diagnostic evaluation after referral.

The odds of a dog presented with AKI to be diagnosed with leptospirosis were 8.9 (when including all dogs) to 12.3 (with a more restrictive case definition) times lower when vaccinated with L4 than when not vaccinated. This is in sharp contrast to the absence of association observed with L2 vaccination. These results suggest that vaccination with L4 in the epidemiological context of Switzerland may be a protective factor against the disease. The retrospective case‐control study design does not however prove a causal relationship, and it is limited to documenting an association. For rare diseases such as leptospirosis however, a prospective vaccine effectiveness study under field conditions would require a very large group of dogs and long‐term observation. Typical experimental models on the other hand fail to reproduce relevant aspects of the naturally occurring disease, including the presence of undetected conditions at the time of vaccination, a wide range of ages, the presence of various comorbidities, and possible exposure to leptospires in the environment.

Because only 1 quadrivalent antileptospiral vaccine (Nobivac Lepto 6, MSD Animal Health) was licensed on the Swiss market until mid‐2017, all study dogs vaccinated with L4 were vaccinated with this specific vaccine. Although newer quadrivalent vaccines show a similar serogroup profile, the vaccinal serovars and strains are different, and extrapolation to other vaccines cannot be made based on the available data.

The numerous cases of leptospirosis in L2‐vaccinated dogs were probably caused mostly by a mismatch between vaccine and infecting serogroups,^11^ although the identity of the true infecting serogroups in Switzerland has not been confirmed yet. The epidemiological data still rely mostly on serological evidence and not on identification of the infecting serovar after isolation from clinical cases.^4^, ^22^, ^23^ The absence of an obvious clustering of cases in any of the time periods postvaccination (Figure 4) suggests that these cases were not associated with an insufficient duration of vaccine protection. The cases of leptospirosis in L4‐vaccinated dogs also could reflect a partial mismatch between vaccine and infecting strains, a broader range of infecting serogroups than previously observed and suspected, or health factors negatively influencing the protective immune response of the dogs at the time of vaccination. Further technical variables including inappropriate injection technique and vaccine distribution and storage can never be ruled out in a retrospective clinical study. The similarities in the disease manifestations, their severity, and the suspected serogroups implied in the cases of leptospirosis in L4‐vaccinated dogs compared to the other dogs with leptospirosis suggest that these dogs did not develop a milder or different form of disease but behaved mostly like unvaccinated dogs. Currently, no evidence exists for the re‐emergence of newer leptospiral serovars not covered by the vaccine, although this cannot be excluded because serological results only poorly represent the true infecting serovar.^24^ Because 87% of the cases of leptospirosis in L4‐vaccinated dogs were observed during the year after initial vaccination, inactivation of vaccine antigens by maternal antibodies and insufficient vaccine response cannot be excluded in some dogs, possibly warranting a modification of the initial vaccination recommendations. Any such change should however first be evaluated in a specifically designed prospective study because the proportion of dogs in the first year of vaccination is disproportionately high in the first years after the introduction of a new vaccine and could bias these results.

One important limitation of our study is its retrospective nature with inherent lack of a standardized diagnostic approach and the risk of preferentially selecting severely affected cases. The use of a referral population from a teaching hospital may further limit the applicability of results to the general dog population by selecting cases on the willingness of their owners to pursue more advanced diagnostic and treatment options. However, the inclusion of a control group with similar presentation and severity of disease and the systematic use of established diagnostic protocols should have decreased these risks and affected both groups similarly, therefore minimizing their effect on the respective incidences of infection in the groups. The applicability of these results to milder or subclinical forms of leptospirosis should be evaluated in separate studies.

The choice of a control population very similar to the case population in terms of clinical presentation and disease severity may further limit the generalizability of the findings to the general dog population of interest. However, this choice also may limit the impact of missed confounders, because these would be more likely to affect similar groups in the same way and therefore have less influence on the conclusions of the study. Because antileptospiral vaccines confer mostly serogroup‐specific protection,^10^, ^11^ the efficacy of this vaccine may vary considerably between regions depending on the prevailing serogroups. It therefore remains important to monitor the regional epidemiology of leptospirosis in dogs and adapt strategies accordingly.

The small size of some of the subgroups may have limited the power of the statistical analyses, but this limitation should be placed in the context of the actual prevalence of the disease. Our study includes 1 of the largest groups of well‐characterized dogs with leptospirosis and a control group of dogs with AKI matched in terms of disease severity. Misclassification bias cannot be ruled out, especially in the absence of a systematic diagnostic evaluation with paired MAT serology and PCR in all dogs and with changes in the availability and selection of diagnostic tests over time. However, the extensive and individualized diagnostic evaluation performed in these dogs is expected to have minimized this bias. The use of MAT serology as a central diagnostic tool has only a limited capability for the diagnosis of the infecting serovars in the individual dog.^24^ However, it remains the main tool available for the confirmation of leptospirosis in dogs in a clinical setting.^23^ Its very good performance for the diagnosis of the disease, rather than for definition of the infecting serovar, suggests that the limitations of this test are unlikely to have significantly affected the results of our study.

In conclusion, being vaccinated with L4 was strongly associated with decreased odds to be diagnosed with leptospirosis compared to unvaccinated dogs, suggesting a protective effect against the disease. This finding is in contrast to the lack of association observed for L2‐vaccinated dogs. Considering the level of evidence available at this time, results of our study support use of quadrivalent antileptospiral vaccines as core vaccines for dogs living in areas with a high incidence of leptospirosis caused by the included serogroups.

## CONFLICT OF INTEREST DECLARATION

Financial support from MSD Animal Health was limited to facilitate collection of data and vaccination history by hiring support staff. Data analysis, interpretation and presentation was performed independently by the investigators. The final manuscript was submitted to MSD before submission for preapproval.

## OFF‐LABEL ANTIMICROBIAL DECLARATION

Authors declare no off‐label use of antimicrobials.

## INSTITUTIONAL ANIMAL CARE AND USE COMMITTEE (IACUC) OR OTHER APPROVAL DECLARATION

Authors declare no IACUC or other approval was needed.

## HUMAN ETHICS APPROVAL DECLARATION

Authors declare human ethics approval was not needed for this study.

## Supporting information


**Table S1** Results of the multivariable logistic regression analyses for associations with a diagnosis of AKI‐L vs AKI‐nL as dependent variable. These analyses include only the 333 dogs with a restricted case definition, excluding dogs without laboratory confirmation of the disease.
