# Supplementary material for: Evaluation of changes in the epidemiology of leptospirosis in dogs after introduction of a quadrivalent antileptospiral vaccine in a highly endemic area
Source: J Vet Intern Med. 2020 Oct 26;34(6):2405–17. doi: 10.1111/jvim.15947 (PMC7694862; doi:10.1111/jvim.15947)
Supplement: Supplementary file 1 — Table S1 Results of the multivariable logistic regression analyses for associations with a diagnosis of AKI‐L vs AKI‐nL as dependent variable. These analyses include only the 333 dogs with a restricted case definition, excluding dogs without laboratory confirmation of the disease. [file JVIM-34-2405-s001.pdf]

Table S1: Results of the multivariable logistic regression analyses for associations with a diagnosis of AKI-L vs AKI-nL as dependent variable. These analyses include only the dogs with a restricted case definition, excluding dogs without laboratory confirmation of the disease (333 dogs for the L4 model and 244 dogs for the L2 model).

**Variable** (reference)	**β**	**SE**	**OR**	**95% CI of OR**	**Wald-P**
**L4-vaccination model**					
Intercept	1.78	0.30	5.913	3.302 - 10.588	<0.001
L4-vaccination status (L0)					
L4+	-2.52	0.39	0.081	0.038 - 0.174	<0.001
L4-	-2.40	0.44	0.091	0.038 - 0.214	<0.001
Age	-0.13	0.03	0.880	0.823 - 0.940	<0.001
Neuter status (intact)					
neutered	-0.53	0.26	0.586	0.349 - 0.985	0.044
74.5% dogs correctly classified; ROC-AUC, 0.789<br>Pearson Chi-square 54.1; 11 outliers on Pearson residual analysis					
**L2-vaccination model**					
Intercept	0.73	0.64	2.069	0.590 - 7.252	0.256
L2-vaccination status (L0)					
L2+	0.80	0.66	2.215	0.610 - 8.046	0.227
L2-	0.04	0.66	1.044	0.285 - 3.832	0.948
Age	-0.10	0.04	0.909	0.839 - 0.985	0.019
Neuter status (intact)					
neutered	-0.95	0.32	0.388	0.209 - 0.722	0.003
62.3% dogs correctly classified; ROC-AUC, 0.702<br>Pearson Chi-square 105.3; 3 outliers on Pearson residual analysis					

Additional diagnostics conducted on both models include the linearity check for the continuous variable age. The variable checks confirmed the inclusion of the model variables except for the L2-vaccination status in the L2-model; none of the variable interactions was retained.

Abbreviations: β, regression coefficient; SE, standard error; OR, odds ratio; 95% CI, 95% confidence interval; ROC-AUC, area under the curve of the receiver operating characteristic curve.
